# Synchrony in Psychotherapy: A Review and an Integrative Framework for the Therapeutic Alliance

**DOI:** 10.3389/fpsyg.2016.00862

**Published:** 2016-06-14

**Authors:** Sander L. Koole, Wolfgang Tschacher

**Affiliations:** ^1^Department of Social Psychology, Vrije Universiteit AmsterdamAmsterdam, Netherlands; ^2^University Hospital of Psychiatry Bern, University of BernBern, Switzerland

**Keywords:** interpersonal sychrony, linguistic alignment, co-regulation, inter-brain coupling, interpersonal neural synchronization, interpersonal emotion regulation, implicit emotion regulation

## Abstract

During psychotherapy, patient and therapist tend to spontaneously synchronize their vocal pitch, bodily movements, and even their physiological processes. In the present article, we consider how this pervasive phenomenon may shed new light on the therapeutic relationship– or alliance– and its role within psychotherapy. We first review clinical research on the alliance and the multidisciplinary area of interpersonal synchrony. We then integrate both literatures in the Interpersonal Synchrony (In-Sync) model of psychotherapy. According to the model, the alliance is grounded in the coupling of patient and therapist’s brains. Because brains do not interact directly, movement synchrony may help to establish inter-brain coupling. Inter-brain coupling may provide patient and therapist with access to another’s internal states, which facilitates common understanding and emotional sharing. Over time, these interpersonal exchanges may improve patients’ emotion-regulatory capacities and related therapeutic outcomes. We discuss the empirical assessment of interpersonal synchrony and review preliminary research on synchrony in psychotherapy. Finally, we summarize our main conclusions and consider the broader implications of viewing psychotherapy as the product of two interacting brains.

Psychotherapy is traditionally known as ‘the talking cure’, a term that originates from Bertha Pappenheim, one of the first patients to receive psychotherapeutic treatment (Breuer and Freud, 1895/1995). Patient and therapist undeniably do much talking in modern psychotherapy. Yet, psychotherapy is more than mere talk. Patient and therapist have bodies that interact with each other in space and time. Consequently, patient and therapist do not just communicate through words, but also through their bodily behavior. Indeed, the bodily behavior of patient and therapist tends to become synchronized during psychotherapy, such that they display coupled patterns in vocal pitch (Imel et al., 2014), head movements (Ramseyer and Tschacher, 2014), and whole body movements (Ramseyer and Tschacher, 2011, 2014). Patient and therapist may even literally get under each other’s skin, as evidenced by matching levels of skin conductance (Marci et al., 2007).

The pervasive synchrony between patient and therapist have so far received little attention within mainstream clinical psychology. This seems unfortunate because research outside the clinical domain has shown that synchrony plays a key role in establishing rapport (Vacharkulksemsuk and Fredrickson, 2012), perspective taking (Wheatley et al., 2012), and the development of adaptive emotion-regulation (Feldman, 2007). There are thus strong grounds to suspect that synchrony is highly relevant to psychotherapy. The need to understand non-verbal processes in psychotherapy has become especially urgent now that new technologies make it possible to conduct psychotherapy without face-to-face contact (Newman et al., 2011) and large-scale implementation of these new technologies is at hand (Kazdin and Blase, 2011).

In the present article, we develop a theoretical framework for understanding the role of synchrony in psychotherapy. In the first section, we begin by reviewing prior theory and research on the patient-therapist relationship, or *alliance*. In the second section, we discuss the notion of synchrony and the pervasive influence that it has on interpersonal relationships. In the third section, we integrate the alliance and synchrony literatures. Specifically, we propose the Interpersonal Synchrony (In-Sync) model, a new theoretical model that explains how patient-therapist synchrony may foster the alliance, and thereby, adaptive emotion regulation. We also consider recent advances in the empirical assessment of patient-therapist synchrony and review relevant research. Finally, in the fourth section of this article, we summarize our main conclusions and discuss some of the broader implications of the In-Sync model.

## The Alliance

During psychotherapy, patient and therapist work together in structured sessions to alleviate the patient’s problems. This working together is the alliance, also known as the therapeutic bond, therapeutic relationship, treatment alliance, helping alliance, or working alliance. It seems intuitively obvious that a good alliance should benefit psychotherapy. However, the therapeutic significance of the alliance has been highly debated among clinical psychologists (Elvins and Green, 2008; Horvath et al., 2011; Wampold and Imel, 2015). In this section, we selectively review theories of and research on the alliance. We begin by situating the alliance among the major therapeutic traditions within clinical psychology. Next, we turn to the main empirical findings that have accumulated with regard to the alliance. We conclude by discussing how scientific understanding of the alliance may be further enhanced.

### Conceptualization of the Alliance

There are presently at least 500 psychotherapies within clinical psychology, which share certain formal characteristics (e.g., delivery by a trained therapist, structured sessions), but differ in contents (Prochaska and Norcross, 2013). Because of the large number of psychotherapies, it is convenient to group them into psychoanalytic, humanistic, and cognitive-behavioral traditions (Wampold and Imel, 2015). These three major therapeutic traditions have each contributed in their own way to the modern notion of the alliance (for more details, see Hougaard, 1994).

Notions related to the alliance first arose within the psychoanalytic tradition. Sigmund Freud, the founder of psychoanalysis, recognized that a positive attachment between patient and therapist helps the patient to stay committed to psychotherapy (Freud, 1912, 1913) (see Horvath and Luborsky, 1993). Sterba (1934) later spoke of an “alliance” between the therapist and the rational parts of the patient’s ego, an idea also present in Freud’s later writings (Freud, 1937). The work of Greenson in the 1960s helped to make the alliance a widely used concept within psychoanalysis (Greenson, 1965, 1967). According to Greenson, the alliance reflects the patient’s motivation and capacity to perform psychoanalytic work. The alliance has remained a major focus in contemporary psychoanalytic approaches, which regard the patient-therapist relationship as a bond that can become deeply meaningful and highly emotionally charged for the patient (Shedler, 2010).

The alliance has further been a major interest in the humanistic tradition in psychotherapy, which has developed in the 1950s from the ideas of philosophers such as Kierkegaard, Husserl, and Heidegger (Cain, 2002; Yalom, 2002; Van Deurzen, 2012). The humanistic tradition has mainly held the therapist responsible for the alliance. Particularly influential has been client-centered therapy (Rogers, 1951; Erekson and Lambert, 2015), which suggests that the therapist should relate authentically with the patient, while offering acceptance and empathy for the patient’s perspective. Carl Rogers, the founder of client-centered therapy, believed that the effectiveness of every form of psychotherapy is ultimately due to the therapist’s capacity to form an authentic, accepting, and empathic relationship with the patient (Rogers, 1957).

Compared with the psychoanalytic and humanistic traditions, the alliance has received less attention within the cognitive-behavioral tradition to psychotherapy (for a comprehensive overview, see Dobson, 2009). Although cognitive-behavioral therapists have regarded a good alliance as a precondition for psychotherapy, most of them do not regard the alliance as directly curative. Focusing on the alliance has also been taken as a devaluation of specific therapeutic techniques that are advocated by the cognitive-behavioral tradition, given that the alliance is common to all psychotherapies. However, therapeutic effects of the alliance are by no means incompatible with specific factors, and indeed, the two types of factors are likely to interact in psychotherapy (Tschacher et al., 2014a). Consistent with this, there is a growing consensus in clinical psychology that common and specific factors jointly shape therapeutic outcomes (Hofmann and Barlow, 2014; Laska et al., 2014).

Even though the therapeutic significance of the alliance has not been directly investigated by cognitive-behavioral psychologists, the cognitive-behavioral tradition has had a major influence on the conceptualization of the alliance. Most of this influence occurred indirectly, through the cognitive-behavioral psychologists’ emphasis on objective empirical methods. Psychoanalytic and humanistic notions of the alliance were originally complex and hard to observe empirically. Under the influence of the cognitive-behavioral tradition, the empirically less tractable elements of the alliance have gradually shifted in to the background, whereas empirically observable aspects have been given more weight (for a conceptual geneology of the alliance, see Elvins and Green, 2008).

By becoming more empirically grounded, the alliance has become increasingly a trans-theoretical construct, whose meaning cuts across therapeutic traditions. This trans-theoretical orientation is clearly apparent in the work of Bordin (1979), who merged different theoretical contributions in his general concept of the working alliance, as (1) agreement of goals; (2) assignment of tasks; and (3) the development of a bond between patient and therapist. Bordin saw these features as central to all psychotherapies. The alliance is the most widely endorsed factor that is common among all psychotherapies (Grencavage and Norcross, 1990; Frank and Frank, 1993). Accordingly, the alliance has been a key interest within the psychotherapy integration movement, which seeks to draw together the different psychotherapy traditions (Grawe, 1997, 2007; Norcross and Goldfried, 2005; Stricker and Gold, 2013).

The modern notion of the alliance subsumes all collaborative elements within the therapeutic relationship (Horvath et al., 2011), regardless of how these elements are associated with the patients’ prior interpersonal attachments. Most researchers distinguish between the personal/social-emotional aspects of the alliance and its task-related aspects (Bales, 1950; Bordin, 1979; Hougaard, 1994). Empirically, however, ratings of personal and task alliance tend to be highly correlated (Elvins and Green, 2008). Researchers from different therapeutic traditions have emphasized either the patient’s or the therapist’s contributions to the alliance. However, the latter may be mainly a matter of perspective, given that the alliance emerges from the mutual interactions between patient and therapist (Bordin, 1979; Hougaard, 1994; Tschacher et al., 2015).

### Alliance Research

The alliance is one of the most frequently studied topics within contemporary clinical psychology (Elvins and Green, 2008; Horvath et al., 2011; Wampold and Imel, 2015). Nevertheless, the therapeutic significance of the alliance remains controversial. One important reason for this controversy is that alliance effects do not fit very well into the standard medical model, which has been widely applied to psychotherapy (for an extended discussion, see Wampold and Imel, 2015). In the medical model, the patient suffers from a physical condition that is treated with a cure that is specifically designed toward alleviating this condition. For instance, a patient suffering from a bacterial infection may be treated with antibiotics by her physician. A basic assumption of the medical model is that the effectiveness of a cure is largely independent of the relationship between the patient and the person providing the cure. After all, most bacteria get killed by antibiotics, regardless of who provides them. The medical model hence leaves little, if any, room for a potential curative role of the alliance.

The methodological gold standard of the medical model is the randomized controlled trial, in which patients are randomly assigned to either a treatment that is expected to be active or a control (placebo) treatment that is expected to be inactive (Danziger, 1994; Shapiro and Shapiro, 2000). To the extent that treated patients do better than patients who received the placebo, the treatment is considered effective. The major strength of the randomized controlled trial is that it allows one to determine if a treatment causes patients’ improvements. Unfortunately, the trial method does not easily lend itself to studying alliance effects. The effects of the alliance are typically very broad and cut across specific psychotherapies (Flückiger et al., 2012). This makes it difficult to determine what a plausible placebo treatment without a good alliance would look like. The alliance may even interact with the placebo, given that placebo effects may become enhanced when the treatment provider evokes a strong (rather than weak) alliance with the patient (Kaptchuk et al., 2008). The effects of the alliance thus go beyond the traditional logic of the randomized controlled trial.

Because of the difficulties in applying the trial method to the alliance, almost all research to date on the alliance has been correlational. In most studies, the patient and the therapist (or sometimes an external observer) rate the quality of the alliance on a questionnaire. Various standardized scales exist to this end (for overviews, see Elvins and Green, 2008; Ardito and Rabellino, 2011). For instance, the widely used Working Alliance Scale has items such as “[My therapist] and I understand each other” and “We agree on what is important for me to work on” (Horvath and Greenberg, 1989). A factor-analytic study of three widely used alliance scales found that the core of patients’ view of the alliance consists of being confident in and committed to a process that feels promising and helpful (Hatcher and Barends, 1996). Items relating to goals and tasks emerged as a single factor, and tend to be correlated in other studies as well (Elvins and Green, 2008), suggesting Bordin’s (1979) distinction between goals and tasks may be too strongly drawn.

The relation between the alliance and therapeutic outcomes has been extensively investigated. In a meta-analysis of 190 independent studies, Horvath et al. (2011) found an average correlation of the alliance and outcomes of individual psychotherapy of 0.275. Other meta-analyses have yielded similar correlations (e.g., Martin et al., 2000). It thus appears that prevailing measures of the alliance on average account for about 7% of psychotherapy outcomes. Although the latter relation is statistically modest, it is robust across different kinds of studies (randomized controlled trials or other), different types of psychotherapy (e.g., cognitive-behavior therapies or other), different alliance measures, and different types of outcomes (e.g., specific symptoms or general wellbeing). Moreover, the average effect of the alliance is larger than the effects of other treatment variables such as therapist adherence to treatment manual or therapist competence (Webb et al., 2010).

Because research on the alliance-outcome link has been correlational, the causal direction of this link remains uncertain (for an extended discussion of this point, see DeRubeis et al., 2005). It could be, for instance, that ratings of the alliance reflect how well the therapy has progressed. However, the alliance-outcome link is only slightly reduced (to *r* = 0.25), and still statistically significant, in studies that assessed the alliance during the first few sessions of psychotherapy (Flückiger et al., 2012). The latter pattern suggests that the alliance is more than just the result of therapeutic success.

Another possibility is that the alliance is linked to outcomes because “better” patients more easily form a strong alliance. However, variations in patients’ contribution to the alliance are not linked to better outcomes (Flückiger et al., 2012). By contrast, therapists who form stronger alliances tend to achieve better outcomes with their patients than therapists who form weaker alliances (Baldwin et al., 2007; Del Re et al., 2012). Therapists who achieve better therapeutic outcomes also score higher on a standardized measure of interpersonal skills such as empathy and warmth (Anderson et al., 2009). Overall, empirical findings are consistent with the idea that the alliance is an active ingredient of psychotherapy.

### Taking Alliance Research Further

As we have seen, modern alliance research has achieved important theoretical and empirical progress. Even so, important aspects of the alliance remain incompletely understood and, in some cases, even hardly investigated. One of the greatest challenges is to understand the dynamic interpersonal nature of the alliance. The alliance is more than the sum of the individual contributions of the patient and therapist. Indeed, the alliance emerges from the mutual interactions between patient and therapist, that reciprocally influence each other as the actions of the patient influence the actions of the therapist, which then go on to influence the patient whose actions again influence the therapist, and so on. Theoretical accounts of the alliance should do justice to these interpersonal dynamics, which go the heart of the alliance as a trans-active, relational phenomenon (Bordin, 1979; Hougaard, 1994; Tschacher et al., 2015).

A second aspect that needs to be further developed is the methodology of alliance research. So far, most alliance research has relied on subjective ratings by the patient and the therapist, and sometimes external observers (Elvins and Green, 2008). When research has gone beyond rating scales, it has mainly examined the verbal-linguistic interactions during psychotherapy (e.g., Muntigl et al., 2013). Alliance research has thus focused almost exclusively on the subjective aspects of the alliance that can be directly explicated in the words of the patient and the therapist. However, there are also physical aspects of the alliance that can be observed objectively, such as patient and therapist’s movements, along with their physiological responses (e.g., heart rates), and neurological activations. Measuring these objective, physical aspects of the alliance is often technically difficult, which may be why these kinds of measures have been understudied. Nevertheless, the scientific analysis of the alliance will not be complete until it addresses both the subjective-linguistic and the objective, physical aspects of the alliance (Tschacher et al., 2015).

A third and last aspect that needs to be developed lies in the connections between alliance research and other scientific disciplines. To date, research on the alliance has been essentially a mono-disciplinary enterprise that is conducted exclusively by clinical psychologists. This approach seems overly restrictive, given that the alliance is a multi-faceted phenomenon that has many meaningful relations with topics that are studied in other scientific disciplines. Indeed, several disciplines have made advances that seem potentially relevant to the scientific analysis of the alliance, including relationships science (e.g., Fitzsimons et al., 2015), social-cognitive neuroscience (e.g., Konvalinka and Roepstorff, 2012), cognitive linguistics (Fusaroli et al., 2012), emotion science (Rimé, 2009), and dynamical systems theory (Tschacher et al., 2015). Consequently, alliance research would do well to nurture a more multidisciplinary orientation.

### Outlook

Alliance research has achieved important progress, by conceiving of the alliance as the collaboration between patient and therapist, and by establishing that patients’ and therapists’ reports of the alliance can account for about 7% of psychotherapy outcomes. Still, many basic questions remain about the nature of the alliance. How does the alliance emerge from the mutual interactions between patient and therapist? How is the alliance manifested in body and brain? And what can disciplines outside clinical psychology tell us about the alliance? In what follows, we seek to derive some answers to these questions from the multidisciplinary area of synchrony research.

## Synchrony

The alliance is an interpersonal phenomenon. Principles that govern interpersonal relations are thus clearly relevant to understanding how the alliance works. Among the most basic of these principles is interpersonal synchrony. Whenever people interact, they are inclined to spontaneously synchronize their neural, perceptual, affective, physiological, and behavioral responses (Semin and Cacioppo, 2008; Wheatley et al., 2012; Repp and Su, 2013). This interpersonal synchrony is part of a broader family of synchrony phenomena that occur throughout the natural and life sciences (Pikovsky et al., 2003; Strogatz, 2003). The word “synchrony” derives from the Greek words *syn*, which means the same or common, and *chronos*, which means time. “Synchrony” thus literally means “occurring at the same time”.

In this section, we selectively review synchrony theory and research. We begin by discussing how synchrony is a unifying principle that can explain many different kinds of complex, self-organizing systems, from pendulum clocks to the human brain. After this, we zoom in on interpersonal synchrony. We end this section with significance of interpersonal synchrony for emotion regulation, a topic that is particularly relevant for psychotherapy.

### Synchrony and Self-Organization

Synchrony operates throughout many biological systems. Well-documented examples of synchrony can be found in cell assemblies, morphogenesis, and evolutionary mutation (Kauffman, 1993; Karsenti, 2008). Synchrony is further important in the functioning of neural networks (Haken, 2013). We consider neural synchronization in somewhat more detail because it illustrates how synchrony works in a biological system that is of central interest to psychologists.

The human brain consists of nearly 100 billion neurons that operate in assemblies of functionally specialized regions. The activities of these neural assemblies must somehow be integrated to yield coherent patterns of thoughts, feelings, and behaviors. This large-scale neural integration may be achieved by synchronizing the activity of neural assemblies (Varela et al., 2001). More specifically, activated neural assemblies have the intrinsic property to oscillate electrically in certain rhythms (Herrmann et al., 2015). Because these oscillations modulate neural excitability, neural assemblies communicate most effectively when their oscillations are synchronized (Fries, 2005). Especially oscillatory rhythms in the beta/gamma range (20–80 Hz) may help facilitate communication between distributed neural functions (Varela et al., 2001; Fries et al., 2007; Uhlhaas et al., 2009). Neural synchrony thus appears to play a key role in coordinating brain functions.

Although synchrony is nowadays a major topic in the life sciences, research on synchrony started in the natural sciences. Indeed, first scientific description of synchrony was rendered by Dutch scientist and mathematician Christiaan Huygens in the 17th century (see Pikovsky et al., 2003). Having just patented the first pendulum clock, Huygens was working to adapt its design for ships on the open sea. During one sea trial, he suspended two pendulum clocks with hooks on a wooden beam (Huygens, 1673/1986). Huygens then observed that the motions of each clock became so much in agreement that that they never receded from another and their sounds were always heard simultaneously. He further noted that the agreement between the clocks became quickly reestablished if it was disturbed. Huygens carefully examined this “sympathy of two clocks” and discovered that the pendula communicated their oscillations onto the wooden beam to which they were suspended, which led the pendula to produce exactly contrary swings.

The development of electrical engineering in the 1920s provided a major impetus to synchrony research (Pikovsky et al., 2003). As it turned out, the frequency of a generator can be synchronized by a weak external signal of a slightly different frequency, a principle that became the basis of the modern radio. It gradually became clear that synchronization phenomena are part of a broader class of self-organizing systems, in which order arises from the non-linear interactions between individual parts. The basic principles of self-organization were formulated by Hermann Haken in the 1970s and 1980s, who was initially trying to understand laser light transitions. Haken’s work led to synergetics theory, a mathematical approach to self-organizing systems that has been applied to both non-living and living systems (Haken, 2012).

Haken’s (2012) synergetics theory shows how the unpredictability of complex systems is often greatly reduced by the emergence of order parameters. Notably, there is a circular causality between the order parameters and the individual components of the system: The individual components generate the order parameters that, in turn, determine the behavior of the individual components. Non-linear dynamics can thus explain how synchronous patterns can emerge ‘spontaneously’ (i.e., without a central coordinating agent) within a complex system. The latter has important implications for the study of human behavior, because there is a deeply engrained tendency among lay people and scientists to attribute coherent patterns in social behavior to the intentions or other qualities of the individual person. The emergence of synchronous behavior, however, does not depend on intentions or any other quality of the individuals who are behaving in synchrony. Rather, synchrony arises as a self-organized behavioral pattern from people’s mutual interactions.

### Interpersonal Synchrony

Synchrony emerges in a wide range of social contexts. For instance, synchronous behavior often characterizes the behavior of large groups, ranging from termite nests and schools of fish (Camazine et al., 2001) to highway traffic (Lee et al., 1998). Moreover, synchrony in face-to-face interactions plays a key role in the formation of interpersonal bonds (Feldman, 2007; Wiltermuth and Heath, 2009; Vacharkulksemsuk and Fredrickson, 2012). The latter, interpersonal, forms of synchrony seem most relevant for psychotherapy.

In an early field study, independent judges observed more movement synchrony in videotaped interactions between high school students and their teachers, relative to control videos composed of randomly selected interactions (Bernieri, 1988). These field observations have been corroborated by the results of behavioral experiments using well-defined cognitive-motor tasks, in which participants can move more or less in synchrony with another. Research on interpersonal movement coordination developed out of studies of intrapersonal synchrony (bimanual finger movements), which resulted in the synergetic model of Haken et al. (1985). Across experiments, participants have been found to display a consistent tendency to synchronize their movements, even when they were previously unacquainted (e.g., Oullier et al., 2008; van Ulzen et al., 2008; Varlet et al., 2011).

Synchrony has further been documented in linguistic communication. For instance, people’s breathing patterns during conversation are highly correlated, either negatively (out of phase) or positively (in phase) (Yang, 2007). Breathing is most closely synchronized near turn-taking and periods of simultaneous laughter or speech, indicating that breathing synchrony is closely tied to the communicative process (Warner, 1996; McFarland, 2001). Furthermore, conversants tend to have highly coordinated postural sway and match each other’s eye gaze, even when they cannot see their partner (Shockley et al., 2003; Richardson D.C. et al., 2007; Brown-Schmidt and Tanenhaus, 2008). Finally, people are spontaneously inclined to synchronize their word use, a tendency that occurs not only for content words (what someone is saying) but also for function words (how someone is saying it) (Pickering and Garrod, 2004; Ireland and Pennebaker, 2010).

Though interpersonal synchrony is ubiquitous, it occurs more readily in the context of positive relationships. For example, in the aforementioned field study among teachers and students (Bernieri, 1988), significantly more movement synchrony was observed when teachers and students mutually trusted each other. Likewise, mothers synchronize their movements more with their own children than with unfamiliar children (Bernieri et al., 1988), and couples high on marital satisfaction synchronize more than couples low on marital satisfaction (Julien et al., 2000). In addition, people synchronize more with people with whom they wish to develop positive relationships (Miles et al., 2011), and with people with whom they have self-disclosed (Vacharkulksemsuk and Fredrickson, 2012).

Once interpersonal synchrony emerges, it has important individual and social consequences. Several experiments have shown that leading people to move in synchrony promotes cooperation and helping behavior (Wiltermuth and Heath, 2009; Kirschner and Tomasello, 2010; Valdesolo and DeSteno, 2011). The behavioral effects of synchrony may be partly explained by increases in pro-social motivation, given that moving in synchrony has been found to increase liking, compassion, and rapport with partners (Hove and Risen, 2009; Valdesolo and DeSteno, 2011; Vacharkulksemsuk and Fredrickson, 2012). However, synchrony may do more than merely shift people’s motivational state. A recent study showed that moving in synchrony led participants to display greater perceptual sensitivity to movements, which in turn was associated with greater success in a subsequent joint-action task (Valdesolo et al., 2010). Consequently, interpersonal synchrony may not only increase people’s willingness to coordinate their actions with others, but also their capacity for doing so.

Research has further begun to illuminate the neural bases of interpersonal synchrony. In so-called hyper-scanning studies, researchers have used various techniques (such as electroencephalographs, magnetic resonance imaging, near infrared spectroscopy) to make simultaneous recordings of brain activities while participants are sharing a task (for reviews, see Konvalinka and Roepstorff, 2012; Babiloni and Astolfi, 2014; Hari et al., 2015). The types of shared tasks that so far have been investigated have ranged from simple button presses to interactive games and group discussions. Across studies, a consistent finding is that joint activities lead to interpersonal synchronization of neural activations. For instance, one experiment simultaneously recorded the brain actions of guitarists playing a short melody together (Lindenberger et al., 2009). The results showed that interpersonally coordinated actions (i.e., behavioral synchrony) are preceded and accompanied by interbrain oscillatory couplings in the prefrontal cortices. Other studies have shown that interpersonal neural synchrony is associated with better joint performance (Cui et al., 2012) and more effective communication (Jiang et al., 2015). Although more work is needed, the available findings suggest that interpersonal synchrony at the behavioral level gives rise to interpersonal neural synchronization.

### Interpersonal Synchrony and Emotion Regulation

The term ‘interpersonal synchrony’ seems to suggest that what is synchronized happens entirely between persons, leaving unchanged what happens within the person. In reality, however, interpersonal synchrony continually interacts with the person’s inner regulatory resources. The boundaries between internal and external regulation thus become blurred. Indeed, synchronous activity may actually lead people’s perceptions of the self and the synchronous other to become merged, both at the level of the body and at the conceptual level (Paladino et al., 2010; Mazzurega et al., 2011). Nevertheless, for analytic purposes, it remains useful to distinguish between internal and external regulation, as long as their mutual dependencies are acknowledged.

During early developmental stages, interaction patterns between the child and caregivers set the stage for interpersonal synchrony (see Feldman, 2007, for an overview). Already within the first hours after birth, mothers strategically initiate vocal and tactile stimulation when the child displays an alert state, establishing the first contingency between the infant’s internal state and the caregiver’s behavior. Such maternal stimulation is associated with the onset of non-verbal synchrony between child and mother, and between child and father (Feldman and Eidelman, 2007). Developmentally primary forms of interpersonal synchrony are thus closely coordinated with systems that self-regulate arousal and attention within the child (Feldman, 2006).

By the age of 9 months, the child’s ability to respond to changes in caregiver’s affect results in mutually synchronous affective exchanges in brief episodes of about 10 s (Feldman, 2007). These micro-level affective exchanges play an important role in the development of the child’s capacity for self-regulation, particularly in regulating own emotional states (Tronick, 1989; Hofer, 1995). For instance, one study showed that mutual affect synchrony with the mother when the child was 9 months predicted self-control abilities at age 2 years, even after statistically controlling for temperament, IQ, and maternal style (Feldman et al., 1999). In a related vein, another study found that greater parent–child synchrony predicted better emotion regulation skills at a later point in time over a period of 10 years (Feldman, 2015). The later findings are consistent with the idea that interpersonal synchrony enhances the capacity for emotional self-regulation.

It seems straightforward that interpersonal synchrony regulates children’s emotions during interactions with their caregivers. After all, synchronous interaction is associated with emotional security (Feldman, 2007), which should down-regulate emotional distress. However, interpersonal synchrony also enhances children’s capacity for emotion regulation when their caregivers are physically absent (Feldman, 2015). The latter may occur because interpersonal synchrony leads the self to become more involved in the interaction (Paladino et al., 2010; Pinel et al., 2015). People’s memory for what is associated with the self is considerably better than people’s memory for what is dissociated with the self (Symons and Johnson, 1997). Moreover, affect-regulatory processes may become associated with the self (Kuhl, 2000; Koole and Coenen, 2007). Consequently, interpersonal synchrony may help children to internalize the emotional security that is associated with the relationship with their caregiver.

Although the links between interpersonal synchrony and emotion regulation have been mostly investigated among children, these links are likely to remain important in adulthood. The clearest support for this notion has been found in the domain of close relationships (Butler and Randall, 2013; Ferrer and Helm, 2013; Timmons et al., 2015). People in close relationships are usually attuned to their partner’s emotions, leading to synchronization of emotional responses between relationship partners, or ‘co-regulation’ (Butler and Randall, 2013). Co-regulation is linked to synchronization of non-verbal behavior (Marci and Orr, 2006; Feldman et al., 2011). Synchrony of emotional processes may thus transfer to close relationships in adulthood. Notably, co-regulation entails more than merely matching of each other’s emotional responses, because this may easily lead to escalating arousal levels, or ‘codysregulation’ (Reed et al., 2015). Instead, co-regulation maintains emotional arousal of the dyad around a healthy homeostatic balance (Timmons et al., 2015).

### Outlook

Synchrony, or the temporal coordination of interacting parts, can be observed in complex self-organizing systems throughout the natural and life sciences. A growing number of studies have examined interpersonal synchrony in neural, perceptual-motor, emotional, social, and behavioral processes. This research has achieved important progress, for instance, by showing that interpersonal synchrony may facilitate positive exchanges and enhance adaptive emotion regulation. Nevertheless, the field has remained somewhat scattered. Neural, perceptual-motor, emotional, social, and behavioral forms of synchrony have been studied separately, without considering how they relate to each other and function together in an interpersonal relationship. We consider a potential integration of these sub-processes in the next section, on synchrony in psychotherapy.

## Synchrony in Psychotherapy

As we have seen, synchrony plays a pervasive role in interpersonal relationships. It thus seems likely that interpersonal synchrony extends to the patient-therapist relationship during psychotherapy. To analyze how this may occur, we present the Interpersonal Synchrony (In-Sync) model of psychotherapy, a new framework that combines insights from various literatures, including social-cognitive neuroscience, cognitive linguistics, psychophysiology, developmental science, relationship science, and emotion science. After laying out the In-Sync model, we discuss the empirical assessment of interpersonal synchrony. Finally, we review the available literature on synchrony in psychotherapy on the basis of the In-Sync model.

### Interpersonal Synchrony Model of Psychotherapy

The core idea of the In-Sync model is that the alliance emerges from the coupling of the neural activity of the brains of the patient and therapist. The more tightly patient and therapist’s brains are coupled, the better the alliance. Of course, patient and therapist’s brains do not communicate directly. Their coupling can thus be achieved only indirectly, through the mutual coordination of the patient and therapist’s behavior and experiences. This mutual coordination is achieved through synchronous activities of the patient and therapist. Synchrony thus helps to establish the alliance, which in turn promotes adaptive emotion regulation in the patient, and thereby good therapeutic outcomes.

**FIGURE 1:**
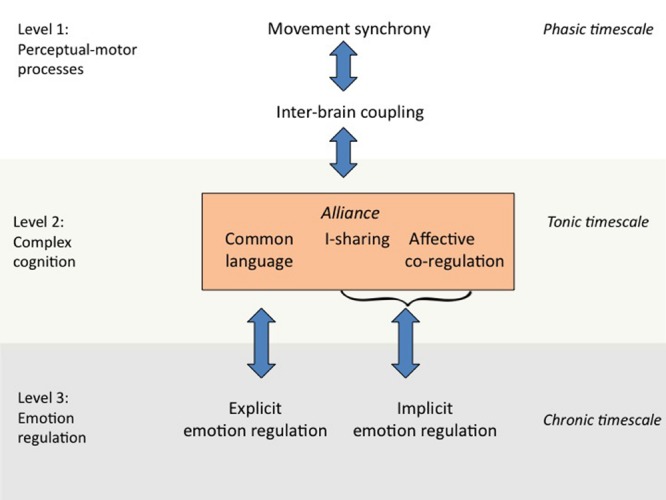
**The Interpersonal Synchrony (In-Sync) model of psychotherapy**.

As can be seen in **Figure 1**, the In-Sync model distinguishes three levels of processing. The different levels are descending in terms of their processing speed and ascending in terms of the complexity of cognitive inferences that are involved. Processes at Level 1 operate on a *phasic* time-scale, which runs from a few hundred milliseconds to about 10 s, and involves the simplest forms of cognitive inferences, namely, automatic associations between perceptions and action. Processes at Level 2 operate on a *tonic* time-scale, which runs from 10 s to about an hour, and involves more complex forms of social cognition, such as language and reasoning. Finally, processes at Level 3 operate on a *chronic* time-scale, which runs from several weeks to years, and involves the development of complex emotion-regulatory abilities. In what follows, we discuss each level in more detail. Notably, there are likely to exist multiple feedback loops between levels, represented in **Figure 1** as double-sided arrows.

Level 1 of the In-Sync model (perceptual-motor processes) starts with movement synchrony, the most basic form of interpersonal synchrony in psychotherapy. Movement synchrony may occur in any perceptual-motor system that can operate automatically, such as facial expressions (Feldman, 2007), eye gaze (Richardson D.C. et al., 2007), breathing patterns (Yang, 2007), or whole-body movements (Ramseyer and Tschacher, 2011). Movement synchrony presumably promotes inter-brain coupling between patient and therapist. The synchronization of motor movements is ideally suited for this purpose because the link between perception and motor action is highly automatic (Prinz, 1990; Dijksterhuis and Bargh, 2001; Wheatley et al., 2012). Motor movements thus provide a continuous stream of behavior that can be rapidly and effortlessly synchronized, even when patient and therapist’s conscious attention is directed elsewhere (Oullier et al., 2008; Varlet et al., 2011).

At Level 2 of the In-Sync model, inter-brain coupling facilitates more complex social-cognitive processes that together constitute the alliance. The key distinction with Level 1 is that cognitive representations at Level 2 no longer have a direct connection with motor systems. Consequently, Level 2 cognition is capable of forming goals and intentions that are maintained over longer periods of time (Goschke and Kuhl, 1993). In addition, Level 2 cognition is capable of retrieving prior autobiographical experiences and connecting these with new experiences in a coherent self-memory system (Conway and Pleydell-Pearce, 2000; Kuhl et al., 2015). Traditionally, the higher cognitions of Level 2 are conceived as separate from the more elementary perceptual-motor processes of Level 1. However, research has shown that complex forms of information processing build upon and extend basic perceptual-motor processes (Barsalou, 2008; Williams et al., 2009; IJzerman and Koole, 2011). In a similar vein, the In-Sync model assumes that the complex cognitions that form the alliance are grounded in elementary perceptual-motor processes.

For analytic purposes, the In-Sync model breaks the alliance down into three different –but closely interacting– component processes. The first component of the alliance is the development of shared mental representations of meanings, that is, a *common language*. The development of a common language occurs through mutual adaptation to another’s linguistic behaviors, a process that is also known as linguistic alignment. Research within cognitive linguistics has shown that more abstract forms of linguistic alignment build upon more basic perceptual-motor processes during face-to-face interaction –the elementary synchronization processes of Level 1 (Pickering and Garrod, 2004). Having a common language facilitates joint problem solving and coordination (Fusaroli et al., 2012). Common language is thus particularly relevant to the task- and goal-related aspects of the alliance (Bordin, 1979).

The second component of the alliance consists of patient and therapist’s mutual sharing of subjective experiences. This process is also known as *I-sharing* (Pinel et al., 2015), after William James’ classic term for the subjective self, the “I”. Experiments have shown that I-sharing promotes social bonding and works as a powerful antidote to feelings of existential isolation (Pinel et al., 2004). I-sharing is therefore most relevant to the personal aspects of the alliance (Bordin, 1979). Synchrony is likely to promote I-sharing, by reinforcing the impression that patient and therapist are undergoing similar experiences (Paladino et al., 2010). Furthermore, to the extent that synchrony fosters the coupling of patient and therapist’s brain states, synchrony may allow patient and therapist to share each other’s experiences (Semin and Cacioppo, 2008).

The third and last component of the alliance is *affective co-regulation* (Butler and Randall, 2013), and consists of the joint regulation of affective responses and their physiological correlates. Co-regulation will often be achieved automatically, through the synchronization of patient and therapist’s motor actions. For instance, when patient and therapist are talking with each other, their breathing patterns will often become synchronized (Warner, 1996; McFarland, 2001), which in turn may synchronize their heart rates and their associated levels of physiological arousal (Hirsch and Bishop, 1981). However, co-regulation entails more than automatic physiological matching. For instance, when a patient gets upset during psychotherapy, it will not be helpful if the therapist becomes similarly upset. Instead, it will be more beneficial if the therapist finds complementary ways of responding to the patient so that they both return to their homeostatic balance. The latter form of co-regulation requires more active regulation, especially on the part of the therapist. Presumably, effective therapists know how to keep the physiological variations during the therapy within healthy homeostatic limits (for a description of experiential-dynamic techniques for co-regulation, see Grecucci et al., 2015). Co-regulation thus appears to be a vital, though largely uncharted, aspect of the alliance.

At Level 3, the therapeutic effects of the alliance lead to improvements in the patient’s self-regulatory capacities. These self-regulatory improvements are likely to apply particularly to the patient’s ability to deal with her or his emotions. The alliance is intimately tied to emotional processes (Greenberg and Safran, 1989). Furthermore, over 75% of the categories of the Diagnostic and Statistical Manual of Mental Disorders (American Psychiatric Association, 2013) are characterized by problems with emotion regulation. Emotion dysregulation thus underlies many of the most common forms of psychopathology, including anxiety and mood disorders (Barlow et al., 2004; Kring and Sloan, 2009; Gratz et al., 2015; Grecucci et al., 2015). The In-Sync model therefore assumes that the therapeutic effects of the alliance are achieved by improving the patient’s capacity for emotion regulation. Notably, the In-Sync model does not rule out that the alliance may also have beneficial effects for the therapist. However, because of the model’s clinical focus, our theoretical emphasis is on the patient’s outcomes.

The In-Sync model further distinguishes between explicit and implicit emotion regulation. *Explicit emotion regulation* is based on self-insight and conscious emotion-regulatory strategies and techniques. Because explicit emotion regulation is mediated by language, it may benefit most from the common language (goal-related) component of the alliance. *Implicit emotion regulation*, by contrast, does not require conscious intentions (Gyurak et al., 2011; Koole and Rothermund, 2011; Koole et al., 2015). The In-Sync model assumes that skills at implicit emotion regulation derive from the combined effects of co-regulation and I-sharing. Through co-regulation, the patient’s physiological arousal becomes stabilized around a healthy homeostatic balance. When co-regulation occurs together with I-sharing, the patient’s self-involvement will be high, which allows the patient to internalize the calming effects of co-regulation. This internalization makes it possible for the patient to implicitly self-regulate similar affective states on subsequent occasions (Kuhl et al., 2015).

### Empirical Assessment of Interpersonal Synchrony

The study of interpersonal synchrony, whether in psychotherapy or other settings, involves a unique set of challenges (see also Delaherche et al., 2012). The first major challenge is to specify in concrete terms what interpersonal synchrony is and what it is not, so that it can be distinguished from other phenomena. Interpersonal synchrony refers to the temporal coordination of behavior between interaction partners. When interaction partners become synchronized, they become adapted to each other’s rhythms and cycles of activity, like people who are dancing together. This mutual adaptation may mean that interaction partners come to display similar behaviors. However, interpersonal synchrony does not always involve imitation or mimicry (Chartrand and Lakin, 2013). For instance, if one interaction partner nods her head in response to another’s hand movements, this still qualifies as interpersonal synchrony. Interpersonal synchrony thus depends on the mutual timing of responses, regardless of the precise form of these responses.

The most commonly used statistical method for assessing interpersonal synchrony relies on determining the correlations between the activities of interaction partners. Researchers first record the activities of each of the interaction partners over time. Most studies of interpersonal synchrony to date have examined movement dynamics, which may be recorded by means of video images or dedicated motion-tracking devices (Delaherche et al., 2012). However, there is growing interest in interpersonal synchrony in physiological responses (Butler and Randall, 2013), and neurological responses (Hari et al., 2015). After the responses have been recorded, their relevant features are extracted and subjected to statistical analysis. Typically, the time series of the interaction partners are analyzed by computing a time-lagged cross-correlation within brief time windows.

The duration of these time windows is a critical factor and may be determined theoretically or through empirical means. Adopting an empirical approach to this matter, one study analyzed videos of 51 same-sex dyads from Stanford University who were engaged in several conversation tasks (e.g., planning a meal together, finding out what they had in common) (Tschacher et al., 2013). The results showed that the dyads’ body movements were significantly associated within time windows of about 6 s. Beyond this time window, the associations between the dyads’ movements were at chance levels. The time window of non-verbal synchrony may represent the ‘social present’, that is, the time duration that interaction partners subjectively experience their togetherness in the here and now. The social present may be akin to the individual present, the time window that people subjectively experience as ‘now’ (Pöppel, 2009).

To determine the time window of interpersonal synchrony, the aforementioned study had to separate genuine synchrony from randomly coinciding movements. This problem applies more generally to synchrony research. Let us say that a patient and a therapist just moved their arm within a second of each other. This could mean that patient and therapist’s movements are indeed synchronized. However, it could also be that patient and therapist independently decided to move their arm and, by a mere stroke of fortune, their individual movements occurred simultaneously. How can we separate synchrony from such chance events? A sophisticated solution to this problem is to construct ‘pseudo-interactions’, that is, artificial datasets of behavior of individuals who did not really interact with each other (Bernieri, 1988). This approach has recently been implemented in automated computer algorithms that can generate pseudo-interactions by randomly sampling from actual interpersonal interactions at very brief time intervals (Ramseyer and Tschacher, 2010). Such stringent statistical controls are necessary to conclude whether interpersonal synchrony has occurred or not.

A final set of challenges derives from the need to record and process activities that become synchronized during interpersonal interactions. Interpersonal synchrony involves a variety of non-verbal responses such as bodily movements, shifts in intonation, or changes in heart rate. Because these non-verbal responses are often subtle and may occur within seconds or mere fractions of seconds, registering them often requires specialized equipment. Fortunately, technological developments have greatly improved the user-friendliness and affordability of the relevant measurement devices. Physiological variables such as heart rate and electrodermal responses can be assessed with ever lighter and smaller devices at increasingly affordable prices (Cacioppo et al., 2007). Likewise, neurological measures have become increasingly non-invasive and adaptable to the investigation of interpersonal dynamics (Hari et al., 2015). These and other technologies have helped to make the assessment of interpersonal synchrony at once more efficient, more accurate, and more comprehensive.

After the data have been recorded, researchers have to extract the relevant features from people’s activities. For instance, in one classic study, judges coded the amount of movement synchrony between students and teachers in frame-by-frame video recordings (Bernieri, 1988). Such manual coding is time-consuming, and typically takes up more time than actual data collection. Again, technological innovation has gone a long way toward addressing this problem. The costs of coding may be considerably reduced if the process can be automated. For instance, researchers at the University of Bern, Switzerland, have developed Motion Energy Analysis (MEA), a software package for automated coding of whole body movements from video images. MEA has become a useful tool for investigating interpersonal synchrony in clinical and non-clinical contexts (e.g., Ramseyer and Tschacher, 2011; Paxton and Dale, 2013). An added advantage is that MEA eliminates the subjectivity of human observers, and thus provides more objective coding. In future years, comparable software will likely become available for the coding of non-verbal affect (e.g., Huis in ‘t Veld et al., 2014; Lewinski, 2015) and vocalizations (e.g., Lee et al., 2014), modalities that currently still rely on manual coding.

### Research on Synchrony in Psychotherapy

Building on the aforementioned technological and methodological advances, recent research has begun to systematically address the role of synchrony in psychotherapy. In the following paragraphs, we review this emerging area. In so doing, we use the In-Sync model as a framework of organizing and interpreting the available findings. For each level of the model, we discuss the extent to which key predictions of the In-Sync model have been supported by empirical findings, have remained unexamined, or when findings appear inconsistent with the model. For each topic, we also note which kinds of research are still needed to fill the gaps in our scientific understanding of synchrony in psychotherapy.

#### Level 1: Movement Synchrony

The first major prediction of the In-Sync model is that patient and therapist should be inclined to synchronize their movements during psychotherapy. A relevant study that examined this issue selected 104 sessions from an archive of videotaped psychotherapies at the outpatient psychotherapy clinic of the University of Bern in Switzerland (Ramseyer and Tschacher, 2011). Patients suffered from a wide range of problems, including anxiety disorders, affective disorders, and other diagnoses except for psychotic disorders and substance dependency. The sessions were analyzed using the automated movement algorithm MEA. The results showed that non-verbal synchrony between patient and therapist was significantly higher than would be expected by chance (i.e., a baseline of pseudo-interactions). Moreover, a reanalysis of a subset of the sample showed that the patient-therapist synchrony occurred both for movements of the head and of the rest of the body, (Ramseyer and Tschacher, 2014). Thus, synchrony in psychotherapy was not only driven by speech activity. Taken together, these findings provide convincing evidence for movement synchrony during psychotherapy.

The In-Sync model further predicts that movement synchrony should facilitate inter-brain coupling between patient and therapist. As far as we know, there have been no studies on this topic. Nevertheless, the link between movement synchrony and inter-brain coupling has been confirmed in motor tasks (Lindenberger et al., 2009). Moreover, inter-brain coupling is higher when conversations partners are facing each other than when they are sitting back-to-back (Jiang et al., 2012), presumably because face-to-face communication allows more movement synchrony. Though research in psychotherapy settings is needed, the available evidence is consistent with the notion that movement synchrony fosters inter-brain coupling.

#### Level 2: The Alliance

The second major prediction of the In-Sync model is that movement synchrony will improve the quality of the alliance. Consistent with this, several experiments that examined simulated psychotherapy sessions have shown that therapists are rated more favorably and as more empathic when they are instructed to make their movements more (rather than less) synchronized with the patient (Trout and Rosenfeld, 1980; Maurer and Tindall, 1983; Sharpley et al., 2001). In addition, the previously discussed clinical study by Ramseyer and Tschacher (2011) found that movement synchrony between patient and therapist, assessed at the start of the psychotherapy, was predictive of the quality of alliance, as rated by the patient at the end of each session. Thus, converging findings support the idea that movement synchrony fosters the alliance.

The aforementioned studies assessed the alliance via subjective reports. However, the In-Sync model also distinguishes objective components of the alliance. These objective components have so far received only little research attention. Nevertheless, we discuss some preliminary work on this topic. The first objective component of the alliance is the emergence of a common language between patient and therapist. One pioneering study of language use during psychotherapy examined 122 sessions by 122 therapists in the USA (Lord et al., 2015). Using written transcripts of the sessions, the study assessed linguistic style synchrony, that is, whether patient and therapist used the same function words (e.g., personal pronouns, prepositions) at each conversational turn. Linguistic style synchrony was significantly correlated with empathy of the therapist, as rated by trained observers in a standardized test. Though preliminary, these findings fit with the In-Sync model’s proposed link between common language and the alliance.

The second objective component of the alliance is I-sharing, or the sharing of subjective experiences between patient and therapist. Given that I-sharing is based on shared subjective experiences, it may not be considered an objective component of the alliance. From the perspective of the In-Sync model, however, shared experiences are closely tied to the interpersonal synchrony. Thus, even though the phenomenological contents of a person’s experience may be subjective, the degree to which the experience is shared can be determined through objective means. Interpersonal synchrony can be assessed with neuro-imaging methods, or inferred from synchrony in movements, language use, or physiological activations. These various forms of interpersonal synchrony are necessary, but not sufficient to conclude that I-sharing has taken place. I-sharing means that the person’s self has become involved in the interpersonal interaction. This self-involvement may be verified by assessing the accessibility of self-related knowledge (Koole and Jostmann, 2004) or memory for self-related material (Baumann and Kuhl, 2003). At present, we are not aware of any research that has used this methodology to examine I-sharing in psychotherapy. The role of I-sharing in the alliance must therefore await future research (see also Pinel et al., 2015).

The third objective component of the alliance is affective co-regulation. To study co-regulation in psychotherapy, researchers need to assess the inter-relations between patient and therapist’s affective responses while they are interacting (Ferrer and Helm, 2013). One study that meets these criteria examined patient-therapist concordance in skin conductance, assessed among 20 patient-therapist dyads in 15-s windows during a 45 min session of psychodynamic therapy (Marci et al., 2007). Skin conductance concordance was associated with higher patient ratings of therapist empathy, and more positive social-emotional interactions for both patients and therapists, as rated by independent observers. These findings suggest that skin conductance concordance may tap into co-regulation processes within the alliance.

Two other studies measured the relation between therapist empathy and patient-therapist synchrony in vocal pitch (Imel et al., 2014; Reich et al., 2014). Vocal pitch synchrony is relevant to affective co-regulation because vocal pitch is associated with emotional arousal (Scherer et al., 2003). One study found that vocal pitch synchrony was positively associated with therapist empathy (Imel et al., 2014). However, the other study found that vocal pitch synchrony was negatively associated with therapist empathy and therapeutic outcomes (Reich et al., 2014). The latter may mean that effective therapists sometimes dampen the patient’s emotions to prevent emotional escalation. Such would be in line with the close relationships literature, where some forms of physiological linkage between partners (e.g., in cortisol levels) are negatively correlated with relationship satisfaction (Timmons et al., 2015). Though more research is needed, these preliminary findings suggest that patient and therapist coordinate their affective responding within psychotherapy. This is consistent with the affective co-regulation within the alliance that is presumed by the In-Sync model.

#### Level 3: Emotion Regulation

The third and last major prediction of the In-Sync model is that patient-therapist synchrony, through its beneficial effects on the alliance, should foster adaptive emotion regulation. The link between movement synchrony and emotion is well-established in parent–child interactions (Feldman, 2007), but has been less investigated in the adult literature. Nevertheless, there are indications that the synchrony-emotion link emerges among adults. One study (Tschacher et al., 2014b) examined the synchrony-emotion link during conversations, a setting that has some similarity with psychotherapy. Specifically, this study recorded movement synchrony and affective changes among 84 previously unacquainted dyads while they were conversing about various pre-selected topics (e.g., tuition fees at the university). The results showed that movement synchrony was associated with increases in positive affect and decreases in negative affect. Moreover, this association was only found after a conversation, consistent with the notion that movement synchrony caused the affective change, rather than the other way around.

Additional findings suggest that synchrony may also foster emotion regulation in clinical settings. In the aforementioned clinical study by Ramseyer and Tschacher (2011), movement synchrony between patient and therapist was a longitudinal predictor of symptom reduction at the end of psychotherapy. Because the majority of patients in this sample suffered from emotional disorders, this finding fits the idea that patient-therapist synchrony fosters emotion regulation. Nevertheless, the evidence is indirect, because psychological symptoms may also become reduced though non-emotion related processes (e.g., more regular sleeping hours, better nutrition). Future work on synchrony in psychotherapy should therefore include more direct measures of patients’ emotion-regulatory skills. In addition, it would be important to assess both implicit and explicit measures of emotion regulation, and to investigate if these show the relations with the different components of the alliance that are proposed by the In-Sync model.

Taken together, research has supported several important aspects of the In-Sync model, particularly for movement synchrony between patient and therapist. At the same time, research on synchrony in psychotherapy is still in an early stage. More well-controlled studies are needed to study the role of synchrony in psychotherapy and to test various predictions of the In-Sync model. In particular, future research should address the effects of synchrony on inter-brain coupling within psychotherapy and on the three objective components of the alliance, common language, I-sharing, and affective co-regulation. Moreover, research should be aimed at the transitions between the different levels of the In-Sync model, to understand how the movement synchrony and inter-brain coupling may become translated into improvements in the alliance and how the alliance may facilitate emotion regulation.

### Outlook

According to the Interpersonal Synchrony (In-Sync) model, movement synchrony supports the alliance –common language, I-sharing, and affective co-regulation between patient and therapist– and thereby facilitates adaptive emotion regulation in the patient. Though research on synchrony in psychotherapy is challenging, recent innovations have enabled rigorous research in this domain. Initial findings are supportive of the In-Sync model, but more research is needed to fully assess the validity of the model.

## Conclusion

In the present article, we have highlighted the role of synchrony in the therapeutic alliance. As the term is used here, synchrony refers to the temporal coordination of the activities of patient and therapist. After reviewing the alliance and synchrony literatures, we integrated both literatures in the Interpersonal Synchrony (In-Sync) model. According to the In-Sync model, synchrony facilitates the alliance, which in turn promotes the patient’s emotion-regulatory skills. Consistent with this, research has shown that patient and therapist synchronize their movements during psychotherapy and that such movement synchrony is positively associated with the alliance and therapeutic outcomes. Moreover, there is suggestive evidence that synchrony plays a role in establishing a common language and affective co-regulation between patient and therapist. The In-Sync model is thus a promising framework for understanding the alliance and its role in psychotherapy.

The In-Sync model builds on and complements prior theory and research on the therapeutic alliance (Horvath and Luborsky, 1993; Elvins and Green, 2008; Wampold and Imel, 2015). In line with this work, the In-Sync model regards the alliance as a collaborative relation between patient and therapist that is important in shaping therapeutic outcomes. The In-Sync model adds a number of new elements, however, including the idea that movement synchrony and inter-brain coupling are foundational to the alliance; a specification of objective components of the alliance, common language, I-sharing, and affective co-regulation; and an emphasis on emotion regulation as a major outcome of alliance effects. Moreover, the In-Sync model introduces a highly multidisciplinary perspective to the alliance, by including insights from social-cognitive neuroscience, cognitive linguistics, psychophysiology, developmental science, relationship science, and emotion science.

More generally, the In-Sync model treats psychotherapy as the product of two interacting brains. This is a fundamentally new perspective because psychotherapy research to date has only considered the patient’s brain as the locus of therapeutic effects (Etkin et al., 2005; Beauregard, 2014; Weingarten and Strauman, 2015). Although the single-brain approach has generated important insights, we believe that it falls short of explaining the dynamic interpersonal aspects of psychotherapy. Ignoring these dynamics denies the inherent interpersonal nature of the alliance, including those aspects of the alliance that are most likely to bring relief from psychological suffering. To fully understand how psychotherapy works, researchers should therefore adopt an inter-brain perspective, by unraveling the interactions between the patient’s and the therapist’s brains.

The In-Sync model further contributes to the interpersonal synchrony literature (Semin and Cacioppo, 2008; Wheatley et al., 2012; Repp and Su, 2013). Because interpersonal synchrony has been studied in various disciplines, findings and paradigms have tended to remain somewhat isolated from each other. For instance, adult research on motor synchrony (Repp and Su, 2013) has so far made little contact with developmental research on synchrony in facial affect (Feldman, 2007), and both lines of research have just started to connect with research on inter-brain coupling (Konvalinka and Roepstorff, 2012) and research on affective co-regulation in close relationships (Butler and Randall, 2013). The In-Sync model helps to draw together these and other lines of research, by using them jointly to analyze the nature of the alliance. In this manner, the alliance may form a center of gravity for interpersonal synchrony researchers, where they can develop and test ideas about the interplay of various forms of synchrony. The resulting insights into the alliance may subsequently be used to understand other kinds of interpersonal exchanges.

The In-Sync model inevitably has limitations. A first limitation is that the In-Sync model assumes the alliance has therapeutic benefits. This assumption seems reasonable given the current state of the psychotherapy literature (Horvath et al., 2011; Wampold and Imel, 2015). Nevertheless, in cases where the alliance has no or only limited benefits, the In-Sync model is not or only partly applicable. A second limitation is the In-Sync model does not include patient expectancies that may give rise to placebo effects, which are part of some models of the alliance (e.g., Wampold and Imel, 2015). Expectancies derive from relatively stable individual beliefs, which are relatively independent from the moment-to-moment synchrony between patient and therapist. Synchrony may influence the patient’s beliefs indirectly, by increasing receptiveness to the therapist’s suggestions (Tanner and Chartrand, 2008; Kelley et al., 2009). However, direct benefits of positive expectancies –placebo effects– cannot be explained by the In-Sync model.

Finally, a third limitation is that the In-Sync model, like all models, is a simplified version of reality. In years to come, research is likely to uncover new factors that shape the effects of synchrony in psychotherapy. For instance, the In-Sync model does not differentiate between whether the therapist is leading or following the patient in their synchronous behavior. Nevertheless, there are preliminary indications that leading versus following in synchrony may have different therapeutic effects (Ramseyer and Tschacher, 2011). If these findings are empirically confirmed, the In-Sync model will have to be extended. In a related vein, models of self-organized systems predict that synchronous actions may fall into one of only two dynamically stable states: inphase or antiphase (Haken et al., 1985). This prediction has been amply confirmed for joint movement coordination (Richardson M.J. et al., 2007; Schmidt and Richardson, 2008). These two modes of behaving in synchrony (Beauregard, 2014) could have differential effects in psychotherapy, but this remains to be investigated in future research. The In-Sync model thus represents a work in progress, which is to be elaborated and revised on the basis of new empirical findings.

Despite these caveats, the In-Sync model has great potential for clinical applications. One possible application lies in improving clinical training programs. Therapists vary substantially in clinical effectiveness, and at least some of these variations are due to their different abilities in forming a strong alliance (Del Re et al., 2012). Improving one’s alliance-building abilities requires accurate feedback, but such feedback is difficult to provide using subjective ratings of the alliance, which are currently standard in the field. The In-Sync model could fill this gap, by fostering the development of objective, standardized measures (e.g., movement synchrony, common language) that can provide valid feedback for therapists regarding their ability to form an alliance with patients. In this manner, the In-Sync model could help therapists to build and strengthen their clinical expertise.

Another possible application of the In-Sync model is in the domain of online psychotherapy. Because the traditional format of face-to-face psychotherapy is time-consuming and expensive, clinicians are increasingly turning to online modes of delivery (Kazdin and Blase, 2011). Online psychotherapy can be effective, especially when it is guided by a trained professional (Andersson and Titov, 2014). However, field studies have shown dropout rates in the range of 75 to 95% (Fleming et al., 2016). One reason for this high dropout may be the reduced physical contact with the therapist during online psychotherapy. From the perspective of the In-Sync model, patient commitment to the therapy and therapeutic effectiveness, may be improved by adding non-verbal modalities to online interventions. For instance, patient and therapist could hold videoconferences. A related option would be to add non-verbal synchronizing modalities to a virtual psychotherapist. There already exist virtual agents with therapist-like functionalities that are capable of responding to people’s non-verbal behavior (DeVault et al., 2014). The In-Sync model could provide a systematic theoretical framework for guiding these developments.

To many, the idea that patients could form a genuine therapeutic relationship with a virtual agent may sound far-fetched. Nevertheless, underneath this heretical idea lies a deeper theoretical insight. As we have seen throughout this article, people appear to be biologically prepared to respond to synchrony in positive, relational terms. This response was already apparent in Huygens’s (1673/1986) description of this synchronized pendulum clocks as having “sympathy” for each other. Consequently, if a virtual therapist can be made to behave in synchrony with patients, patients are likely to respond positively, and may even become attached to it in ways that parallel what clinicians have traditionally called “the alliance”. These notions must currently remain speculative. Nevertheless, we hope that they invite readers to consider the fundamental significance of synchrony in psychotherapy.

## Author Contributions

SK and WT together conceived of the article. SK wrote the first draft and WT made critical revisions. After receiving the reviews, SK drafted the revision and WT made critical revisions.

## Conflict of Interest Statement

The authors declare that the research was conducted in the absence of any commercial or financial relationships that could be construed as a potential conflict of interest.

The reviewer GE and handling Editor declared their shared affiliation, and the handling Editor states that the process nevertheless met the standards of a fair and objective review.
